# Getting a Head Start: Diet, Sub-Adult Growth, and Associative Learning in a Seed-Eating Passerine

**DOI:** 10.1371/journal.pone.0023775

**Published:** 2011-09-19

**Authors:** Kristina M. Bonaparte, Christina Riffle-Yokoi, Nancy Tyler Burley

**Affiliations:** Department of Ecology and Evolutionary Biology, University of California Irvine, Irvine, California, United States of America; University of Lethbridge, Canada

## Abstract

Developmental stress, and individual variation in response to it, can have important fitness consequences. Here we investigated the consequences of variable dietary protein on the duration of growth and associative learning abilities of zebra finches, *Taeniopygia guttata,* which are obligate graminivores. The high-protein conditions that zebra finches would experience in nature when half-ripe seed is available were mimicked by the use of egg protein to supplement mature seed, which is low in protein content. Growth rates and relative body proportions of males reared either on a low-protein diet (mature seed only) or a high-protein diet (seed plus egg) were determined from body size traits (mass, head width, and tarsus) measured at three developmental stages. Birds reared on the high-protein diet were larger in all size traits at all ages, but growth rates of size traits showed no treatment effects. Relative head size of birds reared on the two diets differed from age day 95 onward, with high-diet birds having larger heads in proportion to both tarsus length and body mass. High-diet birds mastered an associative learning task in fewer bouts than those reared on the low-protein diet. In both diet treatments, amount of sub-adult head growth varied directly, and sub-adult mass change varied inversely, with performance on the learning task. Results indicate that small differences in head growth during the sub-adult period can be associated with substantial differences in adult cognitive performance. Contrary to a previous report, we found no evidence for growth compensation among birds on the low-protein diet. These results have implications for the study of vertebrate cognition, developmental stress, and growth compensation.

## Introduction

Conditions experienced during development may have profound influences on the adult phenotype. While the resulting phenotypic variation often reflects adaptive responses to local conditions [1], [2], non-adaptive variation occurs when organisms are stressed by extreme environmental conditions [3], [4] and when traits are under selection for phenotypic canalization [1], [5]. In recent years, evolutionary biologists have devoted increased attention to distinguishing between adaptive and non-adaptive developmental responses [4]–[8] and to understanding fitness consequences of responses to developmental stress, especially those relating to viability [8], [9] and mate-getting [10], [11]. Other important questions include the nature of evolved mechanisms to buffer developing organisms against commonly encountered environmental insults [8], [12]–[15] and causes of individual variation in developmental resilience under stress [16]. Emerging from the various approaches to the topic is the perspective that an individual's ability to regulate its development under stress is an important aspect of its phenotypic quality [8], [9], [11]. Future research advances in this area necessitate identification of developmental priorities and key stressors in taxa under study.

In homeothermic vertebrates, the central nervous system (CNS) appears to be particularly sensitive to a variety of developmental stressors, including poor nutrition [8], [17], [18]. Research on humans [19]–[21] and rodents [22]–[24] indicates that early protein deficits impair adult cognitive function. Specific needs vary among taxa, of course, and the protein requirements of developing humans are likely exceptional [25]. Nevertheless, many organisms may experience variable access to protein at critical points in development and thus would benefit from mechanisms that shield their CNS from deleterious effects of sub-optimal diets. Possible protective mechanisms that have been suggested include the prioritization of CNS development over that of less critical functions [21], and compensatory mechanisms [8], [22]–[23] such as prolonged development when conditions are unfavorable (parallel growth [21]) and variable timing of growth in response to short-term stress (catch-up growth [8], [24]). In fact, however, dietary influences on cognitive capacity of most species are unstudied.

Here we investigated the consequences of variable dietary protein on the duration of growth and associative learning abilities the zebra finch, *Taeniopygia guttata,* an important model of vertebrate neural development [26] and learning [27]. Associative learning is a basic aspect of cognition that may influence success in major life activities [28], so we expect that retaining this cognitive capacity under adverse developmental conditions would be an adaptive priority [29]. Mature grass seed provides relatively little protein and a more restricted range of amino acids compared to that found in half-ripe seed, which has an amino acid profile similar to hen's egg [30]. The availability of half-ripe seed is quite unpredictable in the species' native habitat [31], [32], and the short-lived zebra finch, an opportunistic breeder, will often reproduce on mature seed alone if it is sufficiently abundant [31], [33]; however, pulses of concentrated breeding activity are temporally linked to the availability of half-ripe seed [31], [32].

To maximize the possibility of obtaining diet effects on cognition and to ascertain how such effects correlate with variation in late-development growth patterns, we reared birds on diets high or low in protein and maintained them on their rearing diet well past sexual maturity. Previous research has established that finches reared under conditions closely approximating those we used do vary in adult size, with birds reared on diets low in protein having shorter tarsi and lower mass as a result of lower growth during the nestling phase [34]. Dietary influences on head size and major body proportions have not been reported, however, nor is it known whether low-protein birds show partial compensation for poor nutrition by extending growth of body or brain over a longer time span (parallel growth). A recent study that investigated effects of variable protein during the nestling phase, a period of rapid passerine growth and development, found no overall diet effect on associative learning performance of adult zebra finches [35]. Nevertheless, among birds that experienced low-protein diets as nestlings, an inverse relationship was reported between growth of body mass during the post-fledging period and adult cognitive performance; this pattern was interpreted as demonstrating a cost of catch-up growth following the period of poor nutrition [4], [35]. This set of results formed the basis of our decision to ask whether zebra finches that experience low-protein conditions throughout development differ in performance on an associative learning task from those reared on protein-rich diets.

Birds were cage-reared in family groups, with diet treatments assigned shortly before young hatched. Birds on the low-protein (LO) diet had *ad libitum* access to mature grass seed and several other non-protein resources. Those on the high-protein (HI) diet received the same resources, as well as daily supplements of boiled hens' egg. To minimize possible influences of brood size variation on growth and adult performance, brood size was standardized at 4 by transferring hatchlings under a partial cross-fostering design. We measured 3 aspects of body size (tarsus length, head width, and mass) of sub-adults (55 days of age), young adults (95 days), and mature adults (170 days) and asked whether duration of growth and/or adult body proportions of male subjects was influenced by diet, brood composition and/or the identities of rearing family and family of origin. Finally we asked whether individual variation in growth rate and adult body proportions predicted adult performance on a learning task.

We tested subjects' performance in an associative learning task by asking birds to form an association between an arbitrary stimulus (“yellow curtain”) and a food reward in an apparatus that consisted of a central area with 6 side compartments arranged in a radial design. A fabric curtain at the entrance to each compartment concealed its contents: either an empty seed bowl (5 compartments, each with a black curtain) or a bowl containing seed (one compartment, with a yellow curtain). Each male was individually tested in a series of bouts until he reached the criterion of learning: performance of three bouts in a row in which he entered the yellow-curtained compartment without detouring to explore any black-curtained compartment. Between each bout the location of the rewarded compartment was changed. As a control for the possibility that color preferences are influenced by diet, a subset of the birds was also tested with a black-curtain reward.

## Results

Offspring status as native or cross-fostered did not enter any statistical model, nor did identity of rearing parents.

### Growth of skeletal dimensions and mass

Males reared and maintained on HI diets were larger in all traits (head width, tarsus length, body mass) across all ages, although the diet treatment effect for tarsus length was marginally significant (Table 1). Tarsus length and head width increased with age in both treatments, with no difference in growth rates (means of 1–2 percent in each interval) between treatments (P's>.40). Birds in both treatments failed to show consistent patterns of mass gain after day 55 (Table 1) and no difference in mass change over time intervals (P's>.80). There were no significant effects on trait size resulting from interactions between diet and age (Table 1). Number of females in the rearing brood inversely predicted head width and mass (Table 1).

**Table 1:** Effects of diet treatment, age, and natal brood composition (“sisters”) on size traits of male zebra finches.

Trait	Age	Trait size		Diet effect	Age effect	Sisters effect
		LO	HI			
		Mean±S.E.	Mean±S.E.	Coefficient (P)	Coefficient (P)	Coefficient (P)
Tarsus (mm)^1^				.352± .182 (.054)	.003±.000 (.000)	
	55	13.91±.16	14.34±.15			
	95	14.09±.14	14.38±.14			
	175	14.29±.15	14.60±.13			
Head width (mm)^2^				.396± .083 (.000)	.004±.000 (.000)	−.132±.062 (.032)
	55	12.35±.12	12.58±.07			
	95	12.58±.09	12.92±.07			
	175	12.80±.06	13.06±.07			
Mass (g)^3^				1.130±.442(.011)	−.001±.002(.738)	−.587±.289(.042)
	55	14.79±.28	15.62±.36			
	95	14.92±.32	15.69±.35			
	175	14.96±.33	16.03±.44			

1 Wald Chi^2^ = 84.27, P<0.0001; diet * age interaction ns; 52 subjects nested within 37 families of origin.

2 Wald Chi^2^ = 130.66, P<0.0001; diet * age interaction ns; 52 subjects nested within 37 families of origin.

3 Wald Chi^2^ = 25.57, P<0.0001; diet * age interaction ns; 52 subjects nested within 37 families of origin.

Tarsus growth of individuals was negatively correlated between the sub-adult and adult intervals (Pearson r = −0.611, N = 46, P<0.001 after sequential Bonferroni correction), but all other growth measures varied independently.

**Figure 1:**
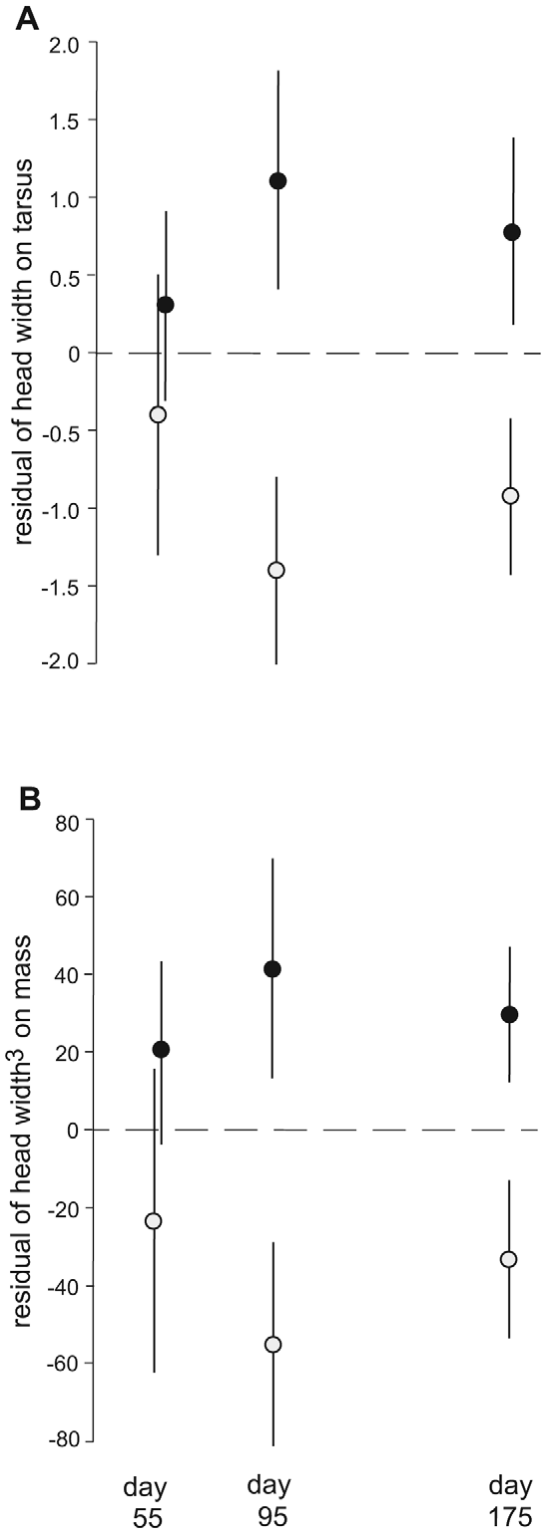
Relative head widths (mean±S.E.) of birds reared on HI vs LO diets. A: residuals of head width on tarsus; B: residuals of head width^3^ on mass. White circles – LO diet birds; black circles – HI diet birds. (See also Table 2.)

Body proportions were assessed as the residuals of mass and head width on tarsus, and head width on mass. In models that considered two-way effects of diet and age, HI diet males had larger (more positive) residuals for all traits measured (Table 2); neither age nor the interaction between age and diet contributed significantly to these models (P's>0.4). At day 55, however, birds in the two treatments did not differ in relative head width, whether assessed as the residual of head width on tarsus length or the residual of the cube of head width on mass (P's>0.4); rather, treatment differences in these body proportions appeared during the late sub-adult interval (Figure 1). Treatment differences in the residual of mass on tarsus were evident from day 55 onward and varied inversely with number of females in the rearing brood (Table 2).

**Table 2:** Effects of diet and natal brood composition (“sisters”) on residuals of male body proportions.

Trait	Model P^1^	Diet effect	Sisters effect
		Coefficient (P)	Coefficient (P)
Mass/tarsus^3^	Wald Chi^2^ = 14.85, P = 0.0006	.844 ±.265(.001)	−.610±.203(.003)
(head width) ^3^/mass	Wald Chi^2^ = 7.68, P = 0.0056	.202±.073 (.006)	–
head width /tarsus	Wald Chi^2^ = 7.37, P = 0.0066	76.3±28.1(.007)	–

1 52 subjects nested within 37 families of origin.

Endocranial volume was significantly correlated with the cube of head width in a sample of 13 adult males (Pearson's r = 0.84, P = 0.003).

### Performance on associative learning task

The 52 males that completed the learning task were derived from 37 unique sets of genetic parents (“families of origin”) and reared by a total of 25 pairs.

**Figure 2:**
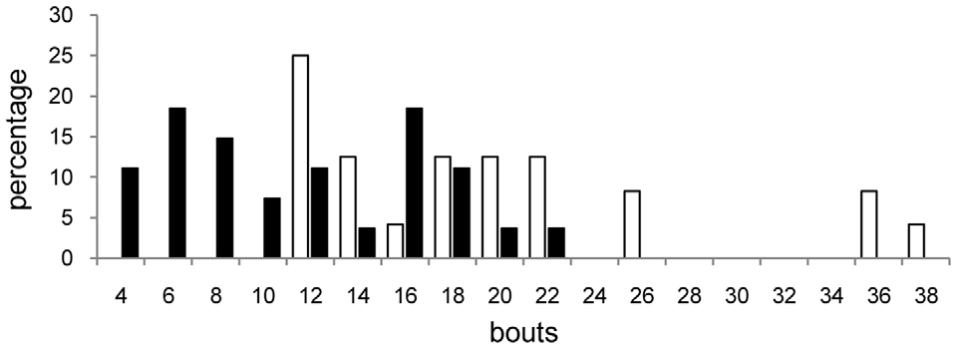
Distribution of number of bouts needed to pass the learning task as a function of diet. (White boxes–LO diet birds(mean±S.E.:20.32±1.57); black boxes–HI diet birds (mean±S.E.: 12.03±1.06). (For statistical effect, see Tables 3 and 4.)

**Figure 3:**
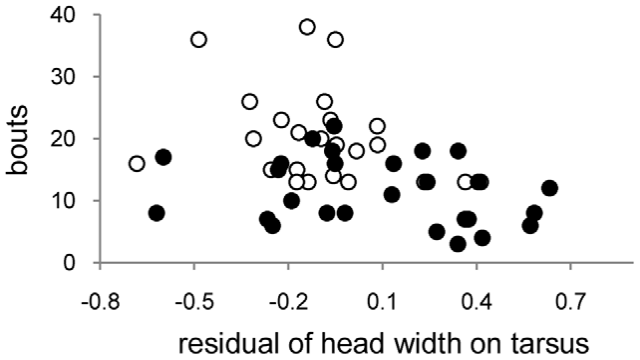
Relationship between residuals of the regression of head width on tarsus (day 175) and bouts taken to pass the learning task. (White circles–LO diet birds; black circles – HI diet birds. For statistical effect, see Table 3. )

Multiple models were run to examine influences on bird performance in a learning task in which a yellow curtain was associated with a food reward. Family-of-origin effects were trivial in these models and are not included (see Methods). The first model considered effects of diet and the residuals of mass and head width at day 175. Diet and the residual of head width on tarsus predicted speed of task mastery (Table 3): LO diet birds required about 70% more bouts to reach the learning criterion (Figure 2), and birds with smaller residuals (small heads in proportion to tarsus length) required more bouts to perform the learning task (Figure 3).

**Table 3:** Effects of diet and head width residual on number of bouts taken to perform the associative learning task.

Variable	Coefficient	S.E.	T	P	β
Diet	−0.979	.229	−4.27	0.000	−.502
Head width residual	−0.815	.376	−2.17	0.035	−.254

Level-one effects only: Model P: F = 15.51 (2,48 df), P<0.0001; adjusted R-squared = 0.367.

**Figure 4:**
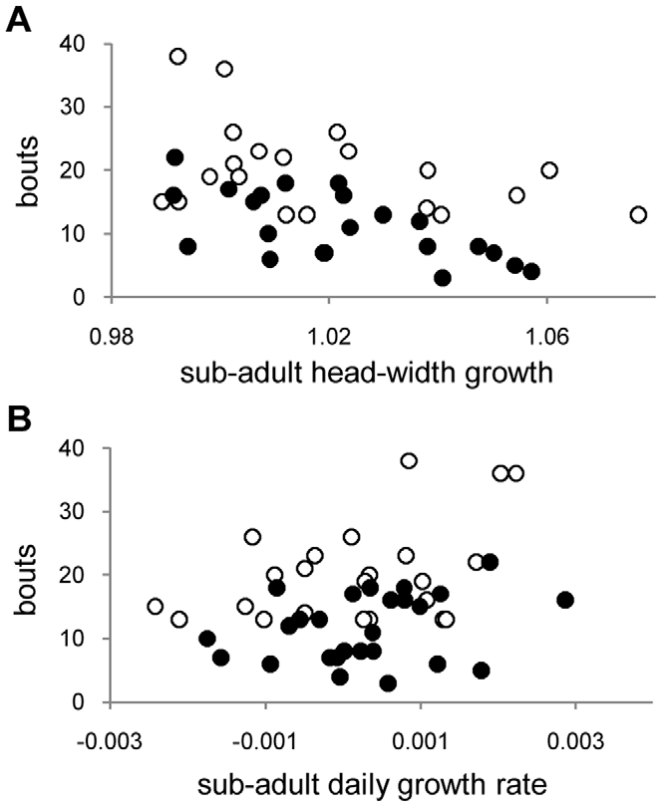
Relationship between early growth (day55 to day 95) and bouts required to pass the learning task. a) head width; b) mass change. (White circles – LO diet birds; black circles – HI diet birds. For statistical effects, see Table 4.)

In the second model, we asked whether variation in sub-adult or adult growth patterns predicted learning score. Two measures of sub-adult growth were found to predict learning score (Table 4): Number of bouts needed to complete the learning task varied inversely with relative head growth between 55 and 95 days (Figure 4a) and directly with relative mass increase during that interval (Figure 4b). (These two traits varied independently [P = 0.92 without Bonferroni correction].) Other growth variables (adult mass increase, adult head growth, and both measures of tarsus growth) failed to predict learning score.

**Table 4:** Effects of early growth variables on number of bouts taken to perform the associative learning task.

Variable	Coefficient	S.E.	T	P	β
Diet	−1.235	.200	−6.17	0.000	−.634
Early head growth	−6.446	1.648	3.91	0.000	−.395
Early mass increase	245.279	87.012	2.82	0.008	.289

Level-one effects only: Model P: F = 20.23 (3,38 df), P<0.0001; adjusted R-squared = 0.615.

In a model that included both sets of independent variables that were significant in previous models (Tables 3 and 4), residual head width at day 175 did not make a significant contribution (P = 0.36). Thus, sub-adult head growth was a stronger predictor of task performance than adult body proportions at the time of testing.

### Effect of reward color on learning performance

**Figure 5:**
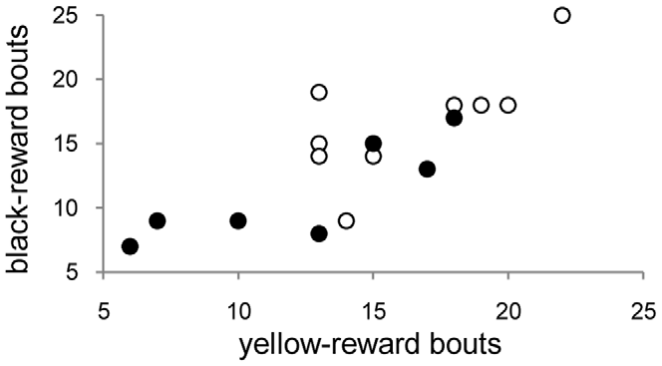
Relationship between bouts required to complete the learning task when yellow versus black curtains were associated with a food reward. (Open circles = LO diet birds; closed circles = HI diet birds. Means±S.E. for black reward: LO –16.67±1.47 (N = 9); HI—11.14 ±1.45 (N = 7).)

For the 16 birds tested with both rewards, learning score on the black-reward test was predicted by learning score on the yellow-reward test (F = 27.02, 1,14 df, P = 0.0001, adjusted R-squared = 0.634; Figure 5); neither the order of testing, nor the interval between the two tests, predicted response (P's>0.30). As in the yellow-reward test, HI and LO birds differed on the number of bouts required to complete the task when black curtains were rewarded (t = 2.62, 14 df, P = 0.02). In sum, treatment effects on learning speed were independent of the color of the rewarded curtain.

## Discussion

Results of this study support a growing consensus that nutritional stress during passerine development has deleterious consequences for the CNS. Prompted by the hypothesis that variation in song quality reveals resilience of males to nutritional stress [36], recent studies have revealed that early food restriction negatively impacts quality of male passerine song [10], [11], [36]–[40]. Working on zebra finches, Spencer and colleagues [39], [40] found that the songs of males which experienced early food restriction were of shorter duration and contained fewer syllables [but see 41,42]; such effects were accompanied by reduction in the size of a key nucleus (HVC) in the song control pathway of the brain of adult males, although other brain areas were not noticeably affected [43]. Nevertheless, a male's singing ability may be indicative of the quality of other phenotypic traits that are also impacted by nutritional or other developmental stressors [11], [36]. Traits that have been hypothesized to be developmentally correlated with song quality include immune system function, spatial cognition, and foraging ability [11]. Recently, Boogert and colleagues [44] reported that the number of syllables in a male zebra finch's song motif predicted the rate at which he mastered a learning task (removing lids to access hidden food). Their study did not include a nutritionally stressed treatment, and their learning task is quite distinct from that investigated here. Nevertheless, the combined results of their study and ours underscore the possibility that cognitive traits that influence mating quality may display developmental correlations with song quality. The breadth and magnitude of developmentally correlated responses to stress is a promising direction for future research on sexual selection [11].

Several additional aspects of the developmental stress hypothesis require greater attention. The idea that song traits reveal heritable resistance to stress [10] underlies much of the current research on the topic, but empirical demonstrations that stress affects song quality fall far short of establishing this connection. An additional problem with this idea is the question of how a female could evaluate “resistance” independent of good information on the degree of developmental stress experienced by the sender [45]. Might not a male that survived in spite of extreme stress have superior genes, albeit impaired song quality, compared to a male that experienced more benign developmental conditions? This consideration suggests that song quality may be more likely to reveal the direct benefits a female can expect to receive from mating with a good songster–such as increased parental care or nest defense–rather than the indirect benefits provided by his “good genes”.

Other important questions include the extent to which a particular signal, such as “number of syllables in a song motif,” reflects specific information about the type of stress endured versus more general information about overall stress levels, and the extent to which stress experienced at different points of development has similar consequences. Most recent studies of diet effects on avian development focus on the nestling phase [4], [36], [46], presumably because this phase is characterized by rapid growth, especially among altricial birds such as passerines [47]. However, in the present study we found that diet effects on head width proportions were absent at the end of the juvenile phase, but emerged during the sub-adult phase (Figure 1). Moreover, our learning test results indicate that sub-adult head growth is functional and suggest that relatively small differences in head growth during the sub-adult phase may have substantial effects on adult cognition (Table 3). That a relationship between head growth and performance on a learning task was found likely reflects the fact that the general learning abilities of birds are centered in regions (nidopallium and mesopallium) of the telencephalon that are large and correlated with overall brain size [26], [48]. Combined with Fisher et al's [35] report that protein limitation during the nestling phase alone does not impact zebra finch performance on the same associative learning task we used, our findings suggest that the sub-adult food environment may have greater effects on some aspects of cognition than environments experienced earlier in development.

We are not proposing, however, that brain growth during the sub-adult phase is uniquely important. Indeed, growth between fledging (approximately day 20) and the beginning of the sub-adult phase is also likely to have cognitive influences, as critical life skills (foraging, predator avoidance) are acquired during this stage. Much further work will be necessary to elucidate consequences of protein limitation at each life stage; to link “head growth” to brain development; and to compare effects of protein limitation with those of other stressors including caloric restriction, the nutritional stressor used in most studies. Despite these limitations, our results suggest that intraspecific comparisons of consequences of relative head/brain growth under different diets may have potential to illuminate evolutionary and developmental trade-offs between the benefits and the costs of developing and maintaining relatively large brains [49] and the cognitive capacities they support [50].

Results of our study salient to the growing literature on developmental flexibility and compensation include our finding that individual performance on the learning task was predicted by sub-adult growth in body mass, with birds that showed greater increase in both treatments taking longer to learn the task. A similar result was reported by Fisher and colleagues [35] for birds that had been shifted from low-protein to high-protein diets after fledging. Their finding has been interpreted as evidence for a cost of catch-up growth to achieve large adult body mass [4], [35]. Our results undermine this interpretation of the pattern, as we found a similar relationship regardless of dietary regime and without providing a stimulus, such as change in diet, to promote a “catch-up” growth spurt. (The relationship between mass growth and learning task performance of birds maintained throughout development on Fisher et al's high-diet treatment was not reported [35].) Moreover, we did not find a correlation between mass change and either measure of sub-adult skeletal growth, and thus no evidence for a direct trade-off among growth variables.

More generally, our collective results lead us to question the assumption that sub-adult acquisition of body mass is likely to be a developmental priority that justifies a trade-off in cognitive function [4], [35], at least for organisms not threatened with imminent starvation. In agreement with the few prior studies on developmental compensation of avian skeletal growth [12], [51], [52], we found no treatment effects on growth of individual size traits, and no significant age-by-diet interactions influencing size traits. In sum, then, we found no evidence for compensation via parallel growth. We suspect that, for birds cage-reared with ample seed availability, absolute body mass may not be the most informative measure of quality. One could even envision a reverse causal explanation for the link between sub-adult mass gain and cognitive performance observed here and by Fisher et al. [35]: Perhaps birds that are slow to complete an associative learning task tend to ingest more when food is readily available to compensate for relatively poor foraging skills.

Finally, our finding that sex composition of broods influenced adult male body size is consistent with field and laboratory results on sex allocation in this species. Field work has revealed sex allocation patterns resulting in female-biased ratios under conditions that promote grass seed development [53], and thus high-protein food conditions. Such sex allocation likely results from sex differences in effects of early diet on fecundity and mating attractiveness of this species [54]–[57]. Because our cross-fostering design manipulated brood sex ratio, and diet treatments were assigned after egg-laying was complete, our results are not confounded by maternal sex allocation tactics at the egg stage. That effects of brood composition on adult male size persisted well into adulthood in a study of caged birds with *ad libitum* access to seed underscores the likely importance of protein limitation in the life history evolution of this seed-eating species. Our research suggests that studies of both developmental compensation and the developmental stress hypothesis would benefit from consideration of maternal effects and effects of brood composition, and that sex differences in developmental stress may inform future studies on sex allocation. The influences of family of origin and brood composition on adult body proportions found here are reminders that the various constraints and trade-offs encountered during development are likely to generate a complex network of effects that will make the unambiguous identification of adaptive, compensatory developmental mechanisms a challenging pursuit [1], [4].

## Methods

All research methods were approved by the University of California Irvine's Institutional Animal Care and Use Committee (IACUC protocol 1334–1998).

### Housing and rearing condition

Breeders assigned to diet treatments were young adults that had hatched in our lab (effective population size>200). These birds had been reared and maintained on a standard (“lab”) diet, which includes *ad libitum* amounts of a commercial mix of mature grass seed, water, cuttlebone, and oyster shell. Supplements of boiled hen's egg and green food were provided three times per week.

After birds selected their mates within colonies of 60 birds, pairs were transferred to cages measuring 66 cm×32 cm×37 cm and given one month to acclimate to the new environment, while remaining on the lab diet. At that time, each pair was provided a nest cup and nesting grass. Seven days after the first egg of a clutch was laid (and after clutch completion), pairs were placed on the diet treatment to which they had been randomly assigned. Families on the LO diet received no supplemental protein. Those on the HI diet initially received 3 g egg daily; the allocation was increased to 5 g when total brood mass reached 10 g, and to 7 g when brood mass reached 20 g. In other respects, the diets of birds in both treatments conformed to the lab diet.

Shortly after hatching, brood composition was standardized to four chicks using a partial cross-fostering design, as adult size varies inversely with brood size in this species [58]. The numbers of males and females surviving to independence (day 35) were recorded. Males were maintained on their natal diet in groups at standard densities throughout the study and were tested for performance on a learning task at about 200 days of age.

### Relative head size and growth measurements

At 55 (sub-adult), 95 (young adult), and 175 (mature adult) days of age (± 3 days), males were measured for mass (to .01 g on an electronic balance), and tarsus length and head width (to .01 mm using electronic calipers). Head width was measured at the mid-point of the cheek patch in line with the opening to the ear canal. One person (CRY), who was not informed of treatment status of subjects, performed all skeletal measurements. Each measurement was taken twice, and values obtained were averaged; when discrepancies greater than 0.10 mm were obtained by this procedure, birds were re-measured. Measurement repeatabilities [59] were calculated for approximately 20 birds at each age by scoring individuals on successive days; all such repeatabilities exceeded 0.93 and were highly significant (p's<.001).

### Associative learning task

Methods closely followed those reported by Fisher et al. [35]. A circular testing arena (32 cm high and 130 cm in diameter) contained six compartments radiating from a central area; the entrance to each compartment was partially blocked by a curtain. Birds were acclimated to the arena for two 48-h sessions, one week apart. During acclimation, all curtains were white, and each compartment contained a bowl of seed. After acclimation, birds were individually tested to determine the number of bouts required to learn the association between curtain color and a food reward. In each bout, only one compartment contained food, and the curtain at the entrance to that compartment was yellow; other compartments contained empty food bowls and had black curtains at their entrance. Between successive bouts, the rewarded, yellow-curtained compartment was changed in a random manner. Each test subject experienced 5 bouts per day until he reached the criterion of learning, which was performance of 3 successive bouts (not necessarily on the same day) with no errors (chance probability = [1/6]^3^ = 0.0046). An error occurred whenever a bird entered a black-curtained compartment before entering the yellow-curtained compartment.

To offset possible short-term effects of diet on performance on the learning task [60], both LO and HI diet birds were maintained on a seed-only diet for at least two weeks before testing. Seed was removed from each test subject's home cage at 1600 h on days before testing. At the start of a day's test (0900 h), a bird was released into a neutral compartment and allowed to recover from handling for 5 minutes; then a door opened, allowing the bird to search throughout 6 compartments of the apparatus. An observer sat quietly in a darkened area behind a screen and recorded the test bird's search path. After the bird discovered the food, he was allowed to feed for 30 s before he was removed from the rewarded compartment. The bird was then rested for 5 minutes while the yellow curtain and food reward were moved to a new compartment. The procedure was then repeated until the bird reached the learning criterion or accumulated 5 bouts. After the day's test was completed, the bird was returned to his home cage and given *ad libitum* access to seed.

As a control for the possibility that birds reared on different diets had different color preferences that influenced their learning speed, a subset of 16 birds was also tested with a black-curtain reward; in this test, the 5 non-rewarded compartments had yellow curtains. For each bird, these two tests were conducted 2–3 months apart. Half the birds were tested first with the yellow reward, while the remainder was tested in the reverse order. Preliminary trials (using different birds) conducted before these tests indicated that zebra finches do not remember the association between curtain color and reward after one month's isolation from test conditions.

### Endocranial volume

To determine if head width measurements reflected endocranial volume, which reflects whole brain size in birds [61], we took head width measurements of 13 males (unrelated to each other), derived from the same laboratory stock, that died during the course of this study and which were over 200 days of age at the time of death. Skulls were cleaned by dermestid beetles and then filled with lead shot to determine their volumes [62].

### Statistical analyses

Linear mixed (REML) models were performed in STATA 9.2. Prior to analyses, variables were transformed as needed to achieve fit of residuals to a normal distribution. Exploratory models indicated that family-of-origin effects were often significant, whereas rearing family effects were not; moreover, nested models including both effects differed trivially from those including only family-of-origin effects. Here we report models including family-of-origin effects unless those effects were so small that STATA could not calculate their standard errors; in such cases, we include only level-one effects. Level-one effects that did not contribute to models were removed using reverse stepwise procedures (P-to-remove = 0.15); offspring status as cross-foster or native was excluded from all models based on this criterion. In all models, LO diet = treatment 0, HI diet = treatment 1.

LMM models that investigated influences on size and growth of traits and trait proportions considered the following level-one variables: diet treatment, age, the interaction between diet and age, and number of females in the rearing brood. Number of males and females per brood varied inversely (Pearson's r = -0.41, N = 52, P = 0.003). To aid interpretation of results, when numbers of females made a significant contribution to a model, we removed this variable to see if number of males made a corresponding effect; however, in no case did number of males per brood approach significance in any model (all P's>0.4).) The first group of models investigated influences on size traits: tarsus length, head width, and body mass. Next, we investigated influences on growth of size traits. Daily growth rates ((log mass_2_ - log mass_1_) /(age_2_ –age_1_)) were computed for mass changes that occurred between days 55 and 95 (“sub-adult growth”) and days 95 and 175 (“adult growth”). Daily growth rates for skeletal measurements could not be suitably transformed for analysis. Instead, these growth estimates were quantified as ratios (age_2_ trait value/age_1_ trait value) and subjected to reciprocal cubic transformation (−1/ratio^3^).

Regression procedures revealed significant linear relationships between head width and tarsus length, the cube of head width and mass, and mass with the cube of tarsus length, at all ages (all P's<.0002). After establishing that residuals of these regressions met assumptions of normality (Shapiro-Wilk test) and homoscedascity (Bresuch-Pagan test), these residuals were used as dependent variables in a third set of LMM models to measure influences on the relative body proportions of subjects.

For the main tests of learning performance, “learning scores” were computed as the square root of the number of bouts required to reach the learning criterion. For the test involving comparison of performance on black- versus yellow-rewards, raw scores (“bouts to complete”) were used, as residuals of raw scores were a better fit in this case. An unpaired t-test was used to compare diet effects on performance on the black-reward test.
